# An attenuated replication-competent chikungunya virus with a fluorescently tagged envelope

**DOI:** 10.1371/journal.pntd.0006693

**Published:** 2018-07-31

**Authors:** Jing Jin, Michael B. Sherman, Daniel Chafets, Nuntana Dinglasan, Kai Lu, Tzong-Hae Lee, Lars-Anders Carlson, Marcus O. Muench, Graham Simmons

**Affiliations:** 1 Blood Systems Research Institute, San Francisco, CA, United States of America; 2 University of California, San Francisco, San Francisco, CA, United States of America; 3 Department of Biochemistry and Molecular Biology, University of Texas Medical Branch, Galveston, TX, United States of America; 4 Department of Medical Biochemistry and Biophysics, Umeå University, Umeå, Sweden; Center for Disease Control and Prevention, UNITED STATES

## Abstract

**Background:**

Chikungunya virus (CHIKV) is the most common alphavirus infecting humans worldwide, causing acute and chronically debilitating arthralgia at a great economic expense.

**Methodology/Principal findings:**

To facilitate our study of CHIKV, we generated a mCherry tagged replication-competent chimeric virus, CHIKV 37997-mCherry. Single particle cryoEM demonstrated icosahedral organization of the chimeric virus and the display of mCherry proteins on virus surface. CHIKV 37997-mCherry is attenuated in both IFNαR knockout and wild-type mice. Strong anti-CHIKV and anti-mCherry antibody responses were induced in CHIKV 37997-mCherry infected mice.

**Conclusions/Significance:**

Our work suggests that chimeric alphaviruses displaying foreign antigen can serve as vaccines against both aphaviruses and other pathogens and diseases.

## Author summary

Chikungunya virus (CHIKV) is an alphavirus capable of causing long term debilitating joint and muscle pain at a great economic expense. Currently there are no licensed vaccines or treatment for CHIKV infection. We generated a modified version of the virus, termed CHIKV 37997-mCherry, stably expressing a fluorescent tag on the surface of the virus. To achieve this, a red fluorescent protein, mCherry, was fused to the virus envelope protein E2. Structural studies demonstrated the presence of mCherry on the virus surface. Infection of mice with CHIKV 37997-mCherry caused less severe disease in the animals compared to wild-type virus. Infection with CHIKV 37997-mCherry induced immune responses against both the mCherry protein and the virus. Furthermore, CHIKV 37997-mCherry is as attenuated as the vaccine strain CHIKV 181/clone 25 in different mouse models, causing less joint swelling and reduced persistence of viral genomes in tissue. Our work suggests that chimeric alphaviruses carrying foreign antigen on virus particles may serve as vaccines against both aphaviruses and other pathogens and diseases.

## Introduction

Chikungunya virus (CHIKV) is a mosquito-transmitted, enveloped positive stranded RNA virus that belongs to the *Alphavirus* genus of the *Togaviridae* family. CHIKV infection causes an acute febrile illness typically accompanied by severe arthralgia, with relapses for weeks to months [1]. In the past decade, CHIKV has spread from endemic areas of Africa and Asia to new populations in Europe and the Americas, making CHIKV a global threat and the most common alphavirus infecting humans. Millions of individuals were infected during the 2000s, resulting in thousands of deaths [2].

The CHIKV RNA genome encodes four nonstructural (nsP1 to nsP4) and five structural (C, E3, E2, 6K and E1) proteins. All five structural proteins are translated as a single polyprotein from which the capsid (C) protein is released via self-cleavage. The envelope polyprotein precursor (E3-E2-6K-E1) is translocated into the endoplasmic reticulum (ER) and processed by host signalase into E1, 6K and E3E2 polyprotein (p62). In the ER, E1 and p62 assemble into heterodimers and subsequently trimerize to form viral spikes. p62 is cleaved by host furin or furin-like proteases into E3 and E2 during trafficking through the Golgi and trans-Golgi network (TGN). The mature CHIKV particles bud at the plasma membrane and have T = 4 quasi-icosahedral symmetry with 240 copies of the E1E2 heterodimer, assembled into 80 spikes on the viral surface; 240 copies of C form an icosahedral nucleocapsid core enclosing viral genomic RNA within the lipid membrane [3]. E1 is a type II membrane fusion protein and sits at the base of the trimeric spike with E2 positioned on top of E1. The ectodomain of E1 consists of three domains [4]. Domain I links distal domain II and membrane proximal domain III. A fusion loop is situated at the distal end of E1 domain II, and is protected by domain B of E2, located at the distal end of E2 [4, 5]. After viral entry into target cells, the acidic environment of endosomes triggers conformational rearrangements within E2, leading to domain B dissociating from the fusion loop [6]. E1 then forms a homotrimer, further exposing the fusion loops of each monomer at the end of the trimeric complex for insertion into the host membrane [7]. E3 limits the movement of E2 domain B until it is cleaved from E2 by furin or furin-like proteases to prevent accidental activation of the fusion peptide before the assembly and budding of mature virions [5, 8]. Studies show alphaviruses can tolerate insertion of foreign antigens to N-terminal of E2 generating chimeric virus presenting foreign antigens [9–11]. There are currently no licensed vaccines or treatments for CHIKV infection. We hypothesize that an attenuated CHIKV carrying foreign antigens may induce immune responses against both CHIKV and the foreign antigen.

In the current study, we report a replication-competent CHIKV with mCherry fused to the N-terminus of E2. The resulting chimeric virus presents mCherry on the virion surface in a repetitive pattern and induces strong antibody responses against both CHIKV and mCherry. The virus is attenuated *in vivo* and provides a safe research tool to study CHIKV virology, as well as demonstrating a useful vaccine platform.

## Methods

### Cell culture, viruses and proteins

Vero and BHK21 cells (ATCC CRL-1586 and CCL-10) were maintained at 37°C in a fully humidified atmosphere with 5% CO_2_ in DMEM (Invitrogen) medium supplemented with penicillin and streptomycin, 10 mM HEPES, non-essential amino acids, and 10% FBS (Hyclone). The CHIKV 37997 and La Reunion (LR) strain virus clone was gift of S. Higgs (Kansas State University) and the CHIKV vaccine strain 181/clone 25 was gift of S. Weaver (University of Texas Medical Branch). Viruses were produced from infectious cDNA clones as previously described [12, 13]. The mCherry gene was inserted between E3 and E2 after the furin cleavage site in CHIKV 37997 genome to make CHIKV 37993-mCherry cDNA clone [14]. mCherry protein fused with 6xHis-tag at the N-terminus was expressed in bacterial cells and affinity purified through Cobalt resins (ThermoFisher Scientific).

### Confocal microscopy

Vero cells were infected with CHIKV 37993-mCherry at a multiplicity of infection (MOI) of 5. At 8 h post infection, the cells were fixed and imaged with a Nikon Ti inverted fluorescence microscope equipped with a Yokogawa CSU-22 spinning disk confocal with a 561 nm laser.

### Gel analysis of purified viruses

Purified viruses were subjected to electrophoresis with 4–12% sodium dodecyl sulfate-polyacrylamide gel (ThermoFisher Scientific) followed by Coomassie staining with SimpleBlue SafeStain (ThermoFisher Scientific) or Western blot analysis with rabbit polyclonal anti-CHIKV 181/25 (IBT Bioservices) and mouse monoclonal anti-mCherry (Sigma).

### Mouse studies

C57BL/6J mice were purchased from The Jackson Laboratory (Sacramento, CA). *Ifnar*^-/-^ mice [15], on a C57BL/6J background, were a gift from Dr. Michael Diamond (Washington University) and bred in house under specific-pathogen-free conditions in microisolator cages (Innovive Inc., San Diego, CA). To study viral pathogenesis in wild type mice, three week-old C57BL/6J mice were inoculated with CHIKV subcutaneously in the left footpad with 10^3^ or 10^6^ PFU of CHIKV in PBS supplemented with 1% heat inactivated FBS. Joint swelling was monitored via left and right foot measurements at the peritarsal region (width x height) using digital calipers. Sera were collected at day 3 after infection and rear ankles were collected at day 7 after infection. To study viral pathogenesis in a lethal mouse model, 6–8 week-old *Ifnar*^-/-^ mice were inoculated with CHIKV subcutaneously in the left footpad with 10, 100 or 1,000 PFU of CHIKV in PBS supplemented with 1% heat inactivated FBS. Joint swelling was monitored via left and right foot measurements at the peritarsal region (width x height) using digital calipers, and mouse survival was monitored for at least 3 weeks. Immunization and challenge with CHIKV were performed by injection of virus subcutaneously in the left footpad. To immunize mice with mCherry, 8 week-old C57BL/6J mice were administered with purified mCherry protein via intraperitoneal injection. Mice were prime immunized with 100 μg of mCherry protein mixed with complete Freund’s adjuvant (Sigma-Aldrich) followed by boosting with 50 μg of mCherry proteins mixed with incomplete Freund’s adjuvant (Sigma-Aldrich) 2 weeks later.

### Ethics statement

All animal experiments were performed with the approval of the Institutional Animal Care and Use Committee at PMI Preclinical, LLC (San Carlos, CA), protocol number IAC 1705. Mice received humane care according to the criteria outlined by the National Research Council’s Institute of Laboratory Animal Resources in the “Guide for the Care and Use of Laboratory Animals”. All injections of mice with virus were performed under anesthesia with isoflurane or ketamine and xyalzine.

### Viral Quantification by qRT-PCR

Viral RNA in culture supernatant or mouse blood was extracted by the QIAamp Viral RNA Mini kit (Qiagen) following the manufacturer’s protocol. To extract viral RNA in mouse tissues, tissues were first lysed in TRIzol (ThermoFisher Scientific) using Qiagen Tissue Lyser II (Qiagen). RNA was extracted following the manufacturer’s protocol. Isolated RNA was analyzed by qRT-PCR and compared to a standard curve generated from CHIKV-GLuc plasmid [16] with the following primers: CKV_For, 5’-TGGCCACCTTTGCAAGCTC-3’; CKV_Rev, 5’-GGGATGAACTCCATTGTAGC-3’; and CKV_Probe, 5’/56-FAM/AGGTACGCACTACAGCTACC/36-TAMSp/3’.

### Enzyme-Linked immunosorbent assay

Anti-CHIKV and anti-mCherry antibodies in mouse sera were quantified using virion-based ELISA. To detect anti-CHIKV antibodies, Immulon 4HBX plates (96-well, Thermo Scientific) were coated with CHIKV 181/25 virus purified through sucrose cushion (2.5 x 10^8^ genome copies/well). To detect anti-mCherry antibodies, Immulon 4HBX plates were coated with purified 6xHis-mCherry protein (1 μg/well). Serial dilutions of mouse serum were added to the plates. Serum of naïve mice was used as the background control. Next, plate-bound antibodies were detected with biotin-conjugated goat anti-mouse IgG antibodies (Southern Biotech), followed with streptavidin-conjugated horseradish peroxidase (Southern Biotech). Binding was detected with 3,3’5,5’-tetramethylbenzidine substrate (Neogen). Endpoint titers were defined as the reciprocal of the last dilution to have an absorbance two times greater than the background control.

### Plaque assay and plaque reduction neutralization tests

Neutralizing activity of mouse serum was quantified using a plaque reduction neutralization test (PRNT). Serial dilutions of mouse serum were incubated with 50 PFUs of challenge virus for 1 hour at 37°C, followed by infection of Vero cells for 1 hour at 37°C. To quantify virus by plaque assay, Vero cells were incubated with serial dilutions of virus containing supernatant for 1 hour at 37°C. Next, cells were overlaid with medium containing 2% FBS and 0.8% agarose, followed by culture at 37°C. Plaques were counted 2 days later.

### Purification of CHIK viruses

To coat ELISA plates, CHIKV 181/25 was produced in BHK21 cells virus and was pelleted through a 20% sucrose cushion. To make gradient-purified viruses for single particle cryoEM study, we adapted a published protocol [17]. CHIKV 37997 and CHIKV 37997-mCherry viruses were produced in BHK21 cells and first concentrated by pelleting the culture supernatant through a 20% sucrose cushion. Then, the resuspended virus was layered onto 2 ml of 60% OptiPrep (Sigma-Aldrich) and spun at 50,000 *x g* for 1.5 hour using a SW28 rotor. After ultracentrifugation, the supernatants were removed to leave 4 ml above the virus band. The remaining 4 ml supernatant, the virus band and the underlay of 2 ml of 60% OptiPrep were mixed to reach a final concentration of 20% OptiPrep. The mixture was spun at 360,000 x g for 3.5 hours with a NVT65.2 rotor. The virus band was extracted and buffer exchanged to NTE buffer (20 mM Tris, pH 8.0, 120 mM NaCl, 1 mM EDTA) using an Amicon Ultra-2 Centrifugal Filter Unit with Ultracel-100 membrane (Millipore).

### CryoEM reconstruction of CHIKV 37997-mCherry

Purified CHIKV 37997-mCherry virus was flash-frozen on C-Flat copper grids (R2/2, 200 mesh) in liquid ethane. The movie-mode data of CHIKV 37997-mCherry was taken on a DE-20 direct electron detector (Direct Electron, LP, San Diego, CA) on a 200-kV JEOL 2200FS microscope under low-dose conditions. We used a frame rate of 25 frames/s and 1.52 s exposure that corresponded to ∼38 e/Å^2^ dose/image. Individual frames in each image were aligned using DE_process_frames.py script provided by Direct Electron (Direct Electron, LP, San Diego, CA) using radiation damage compensation to increase low-frequency content in the final images, thus facilitating subsequent particle alignment. Particle images were automatically boxed out using the E2BOXER program from the EMAN2 suite [18]. EMAN2 was used for CTF determination and correction; the subsequent processing (particle alignment, orientation search and refinement, and 3D reconstruction) was done in IMAGIC-5 [19].

### Statistical analyses

All data were analyzed using Prism software (La Jolla, CA) and statistical significance was assigned when *P* values were < 0.05. Neutralization curves were calculated using non-linear regression. Mouse survival curves were compared with Gehan-Breslow-Wilcoxon test. Viral titers and mouse joint swelling were analyzed using a one-way or two-way ANOVA test.

## Results

### Generation of a replication-competent CHIKV with fluorescently labeled envelope glycoprotein

**Fig 1:**
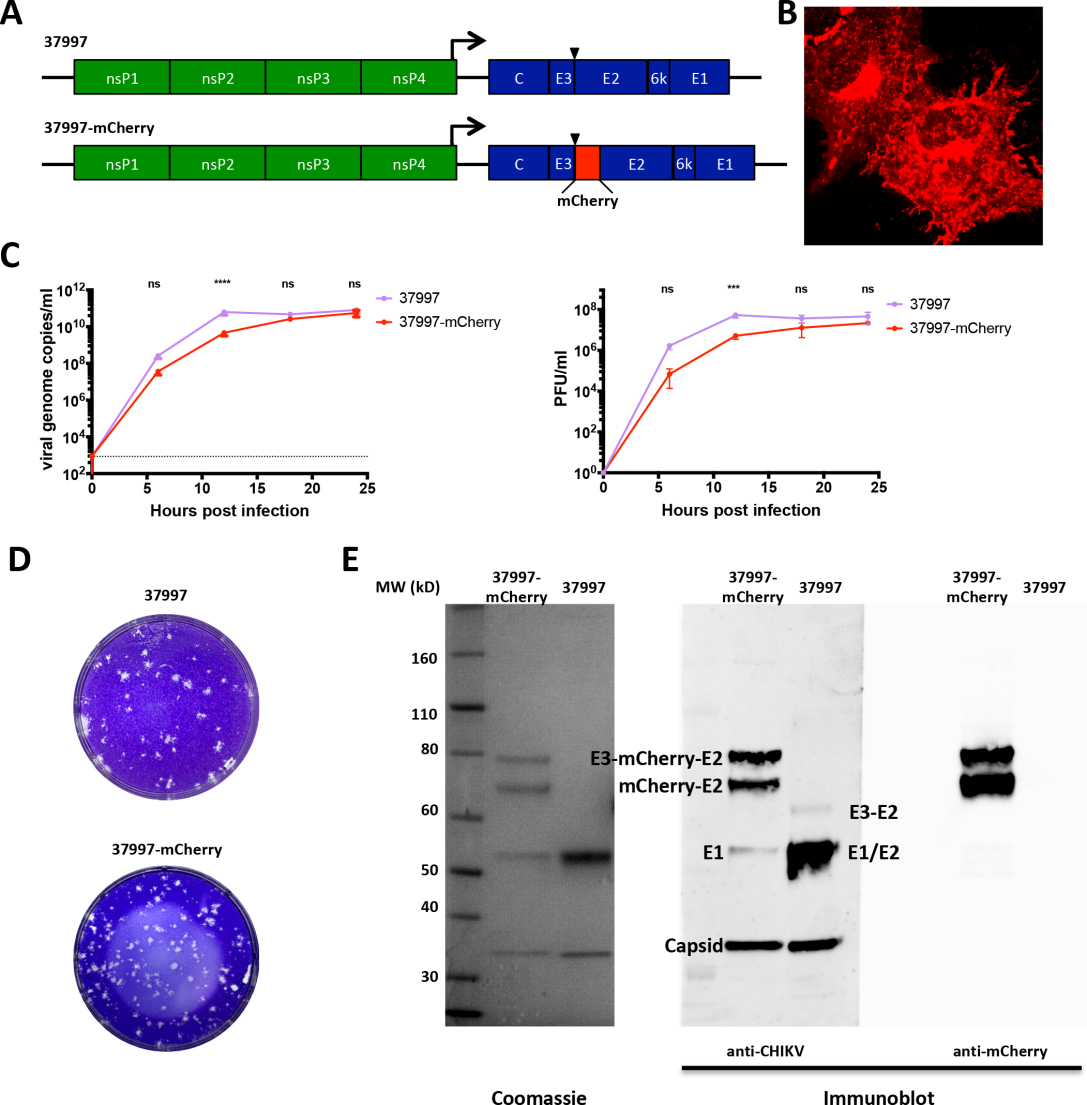
Generation of a replication-competent chimeric CHIKV 37997-mCherry. **(A)** Schematic illustration of the viral genomes of CHIKV 37997 *vs* CHIKV 37997-mCherry. **(B)** Localization of mCherry-E2 in CHIKV 37997-mCherry infected cells. Vero cells were infected with CHIKV 37997-mCherry and imaged with confocal fluorescence microscope. **(C)** Viral growth curves of CHIKV 37997 *vs* CHIKV 37997-mCherry. BHK21 cells were infected with CHIKV 37997 or CHIKV 37997-mCherry at a Multiplicity of Infection (MOI) 0.1. Virus release at indicated time points post-infection was measured by qRT-PCR analysis of viral genomic RNA and plaque assay of infectious viral particles in culture supernatant. The dashed line indicates the detection limit. Statistical significance was determined by ANOVA with a Sidak’s multiple comparisons test (***p<0.0002, ****p<0.0001) (n = 3). **(D)** Representative plaques formed by CHIKV 37997 and CHIKV 37997-mCherry on Vero cell monolayer. **(E)** Purified CHIKV 37997-mCherry was separated on a SDS-PAGE gel followed by Coomassie-staining and Western blot. mCherry-E2, E1 and capsid bands are indicated.

To make a fluorescently tagged CHIKV, we inserted DNA encoding mCherry into a cDNA clone immediately downstream of the furin cleavage site between E3 and E2 in CHIKV 37997 (**Fig 1A**). The resulting chimeric CHIKV 37997-mCherry virus was then produced as previously described [16]. In CHIKV 37997-mCherry infected cells, mCherry-E2 displayed a typical glycoprotein expression pattern: from Golgi apparatus to endosomes to the plasma membrane, and alphavirus-induced Env positive membrane extensions were observed (**Fig 1B**). Next, we compared viral growth curves of CHIKV 37997-mCherry with the parental CHIKV 37997 virus *in vitro*. Viruses released in the culture supernatants were quantified by viral RNA level measured by qRT-PCR and infectious particle number measured by plaque assay. In BHK21 cells, CHIKV 37997-mCherry demonstrated slightly slower growth kinetics compared to parental wild-type virus at early time points (before 12 hours post-infection), however no differences were seen at later time points (after 18 hours post-infection) (**Fig 1C**). CHIKV 37997-mCherry formed plaques similar to parental viral plaques (**Fig 1D**) and had a similar viral genome copy to PFU ratio (2.49 x 10^3^) as the parental CHIKV 37997 (2.75 x 10^3^). In gradient purified CHIKV 37997-mCherry virus E1 and capsid proteins were present at the expected size (**Fig 1E**). The double bands that react with both anti-mCherry monoclonal antibody and anti-CHIKV polyclonal antibody are probably E3-mCherry-E2 precursor and mCherry-E2 fusion proteins. Insertion of mCherry between E3 and E2 might slow down the furin cleavage of E3 because the parental CHIKV 37997 virus contains very little uncleaved E3-E2 (p62) precursor. The smaller sizes of E3-mCherry-E2 and mCherry-E2 than expected may due to some glycosylation difference with mCherry insertion rather than truncation within the fusion protein as the viral RNA genome was intact in gradient purified CHIKV 37997-mCherry (**S1 Fig**).

### Presentation of mCherry at the surface of CHIKV 37997-mCherry virions

**Fig 2:**
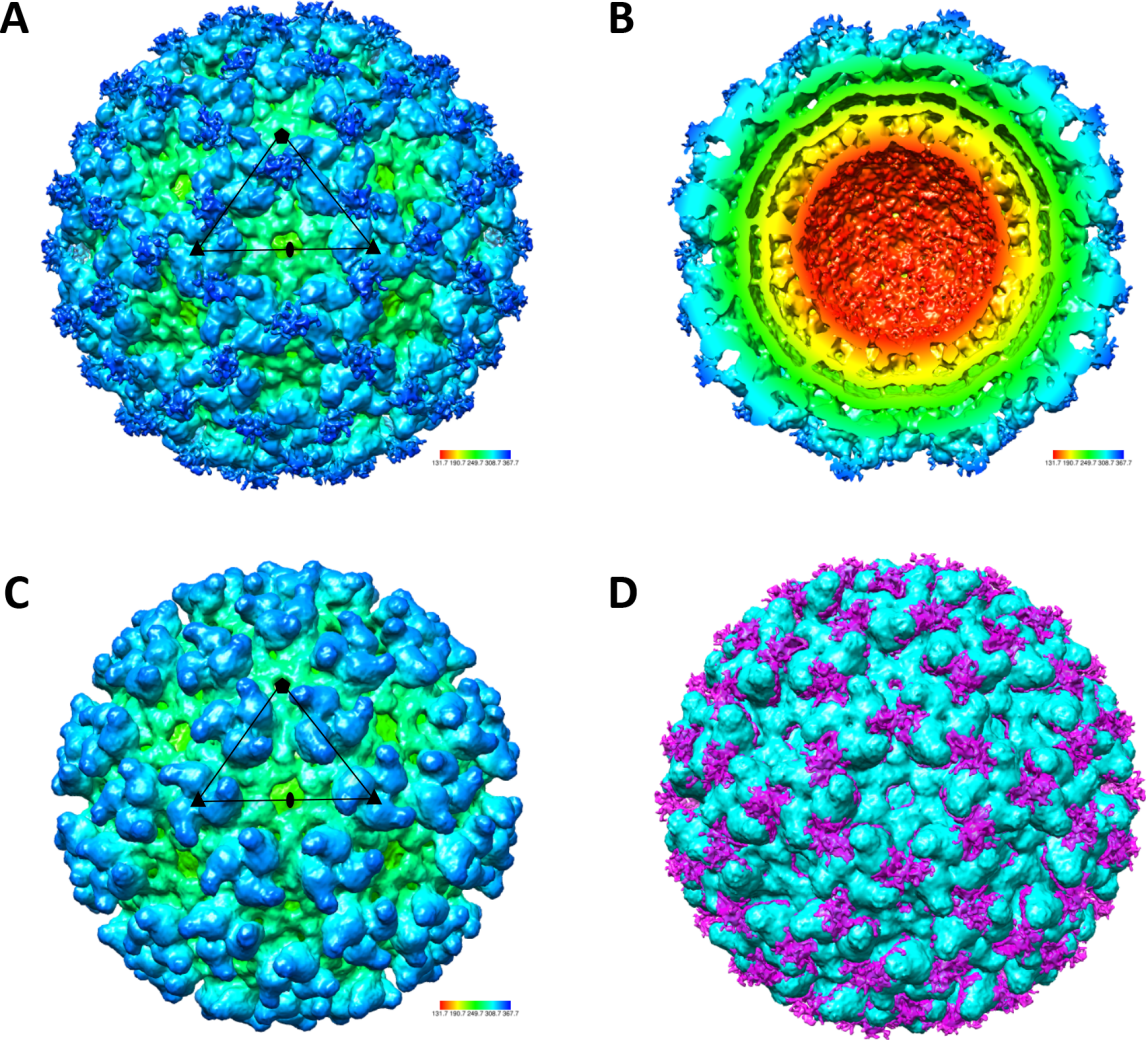
Presentation of mCherry on surface of virus particles. Single-particle cryoEM reconstructions of CHIKV 37997-mCherry (**A, B & D**) and CHIK virus like particle (VLP) (**C**). (**A**) Surface-shaded and (**B**) cross-section views of CHIKV 37997-mCherry density map show a T = 4 icosahedral structure. (**D**) The comparison between CHIKV 37997-mCherry and CHIK VLP density maps reveals mCherry proteins on virus surface that are labeled in magenta.

To understand the presentation of tagged mCherry proteins on chimeric CHIKV 37997-mCherry virus, we performed single-particle cryoEM analysis of purified CHIKV 37997-mCherry virus particles (**Fig 2**). CHIKV 37997-mCherry virions display a typical T = 4 icosahedral structure similar to wild-type CHIKV and other alphaviruses (**Fig 2A and 2B**). Compared with the cryoEM map of CHIK virus-like particles that we previously obtained (**Fig 2C**)[16], CHIKV 37997-mCherry displayed extra density on the particle surface representing mCherry proteins fused at the N-terminal of E2 (**Fig 2D**). The mCherry proteins were well exposed, occupying the space between viral spikes on the particle surface (**Fig 2D**).

### CHIKV 37997-mCherry is attenuated in immunocompromised mice

**Fig 3:**
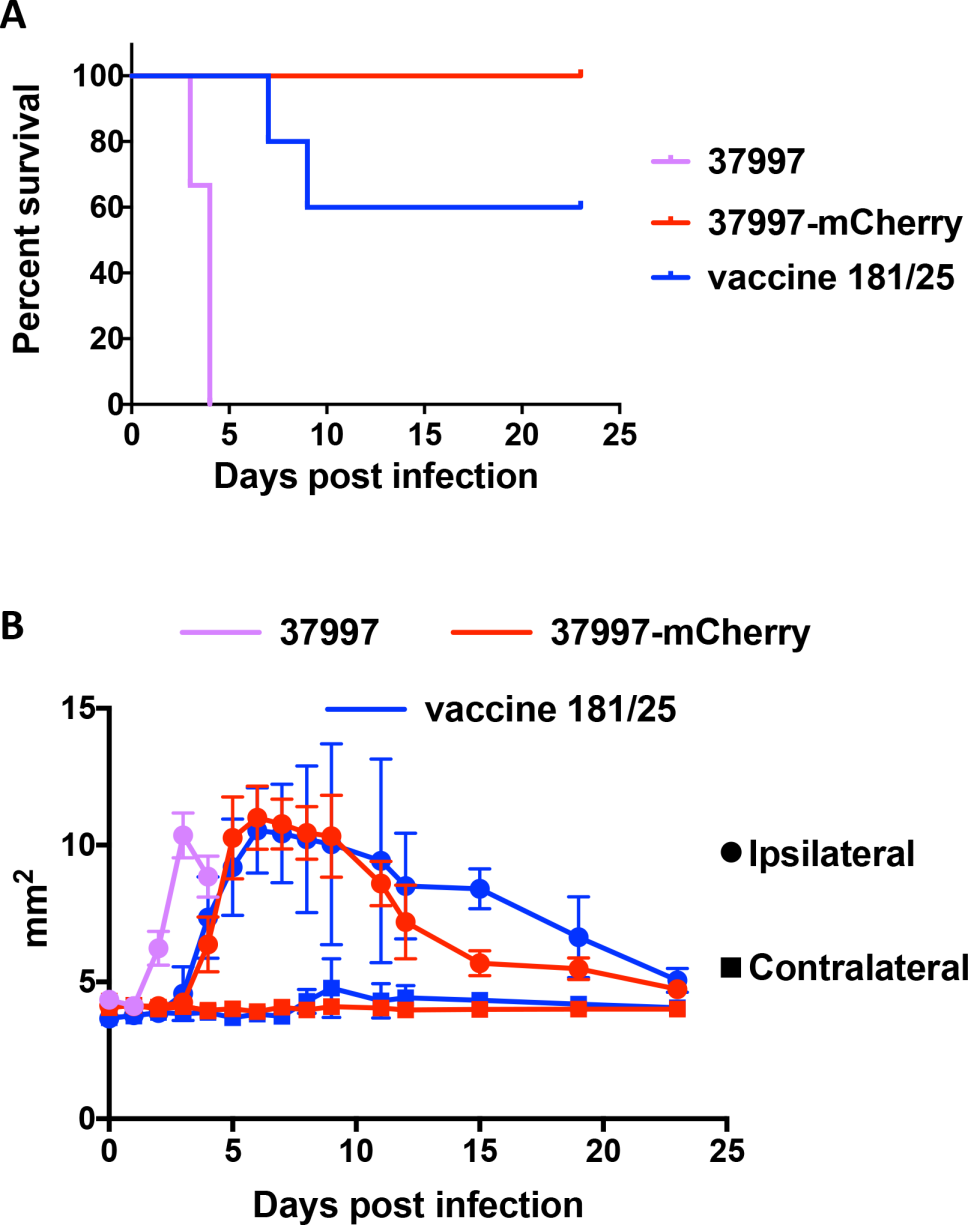
Attenuation of CHIKV 37997-mCherry in immunocompromised mice. IFNαR-/- mice (5 per group) were injected with 10 PFU of indicated viruses in the left rear footpad. (A) survival of mice was monitored, and (B) Left (circles) and right (squares) rear footpad sizes (width x height) were measured for 4 weeks. Data shown are pooled results of three repeated experiments.

After we confirmed that CHIKV 37997-mCherry replicated to a similar level as wild-type CHIKV 37997 *in vitro*, we next compared their growth and pathogenesis *in vivo*. In an IFNαR-/- lethal infection model, injection of 10 PFU of CHIKV 37997 into the mouse footpad resulted in death in 100% of the mice in 4 days. In contrast, all the mice infected with 10 PFU of CHIKV 37997-mCherry survived through the period of observation (23 days) (**Fig 3A**). Surprisingly, infection with the CHIKV vaccine strain 181/clone 25 (CHIKV 181/25) also resulted in death in 40% of the mice within 9 days (**Fig 3A**). Infection of all three strains caused footpad swelling on the ipsilateral side, but with a delay observed with CHIKV 37997-mCherry and CHIKV 181/25 infections compared to CHIKV 37997 (**Fig 3B**).

**Fig 4:**
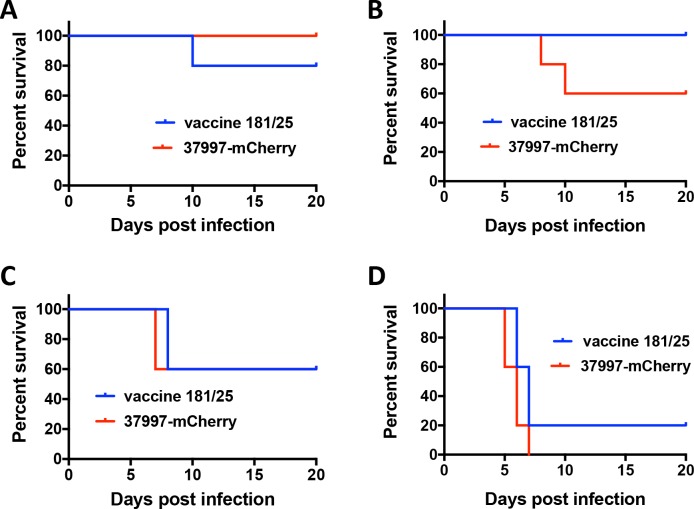
Pathogenesis comparison between the vaccine strain CHIKV 181/25 and mCherry tagged CHIKV 37997-mCherry. Survival of IFNαR-/- mice was monitored for 3 weeks following inoculation of (A) 10 PFU (B) 10^2^ PFU (C) 10^3^ PFU and (D) 10^4^ PFU of indicated viruses in the left rear footpad (5 mice per group).

Because CHIKV 37997-mCherry was found to be attenuated compared with the parental virus at a 10 PFU dose, we next compared its pathogenesis with the vaccine strain CHIKV 181/25 in IFNαR-/- mice over a range of different inoculation doses. Survival curves of mice infected with 10 PFU to 10^4^ PFU of CHIKV 37997-mCherry *vs* CHIKV 181/25 showed no significant difference (**Fig 4**), indicating CHIKV 37997-mCherry is similarly attenuated as CHIKV 181/25 in IFNαR-/- mice.

### Infection with CHIKV 37997-mCherry induced a strong neutralizing antibody response and provided protection against pathogenic CHIKV infection

**Fig 5:**
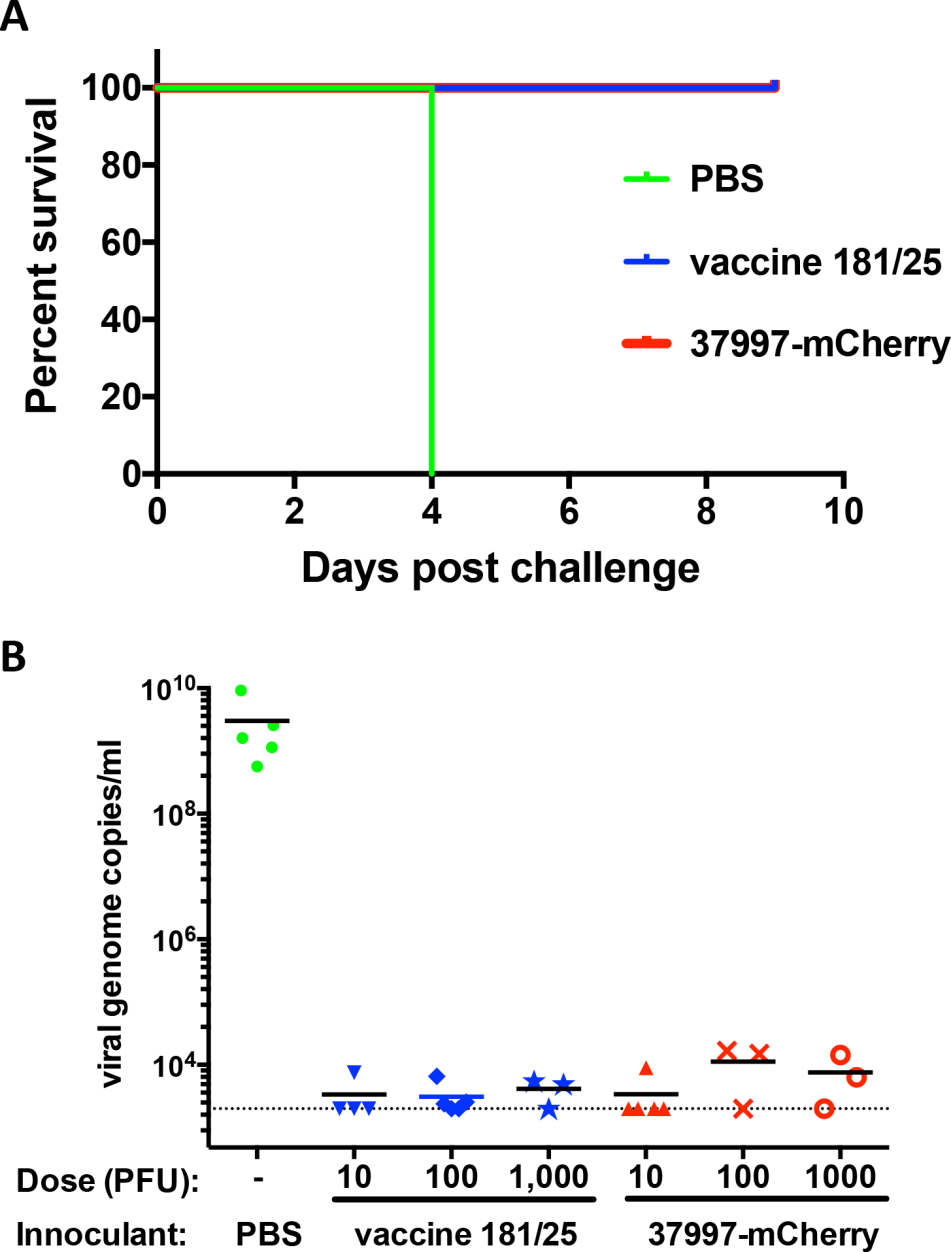
CHIKV 181/25 and CHIKV 37997-mCherry protected IFNαR-/- mice from lethal pathogenesis of CHIKV LR. IFNαR-/- mice that survived CHIKV 181/25 (4 mice survived 10 PFU, 5 mice survived 100 PFU and 3 mice survived 1,000 PFU) and CHIKV 37997-mCherry (5 mice survived 10 PFU, 3 mice survived 100 PFU and 3 mice survived 1,000 PFU) infection were challenged with 10 PFU of CHIKV LR at three weeks post-infection. Mice without previous infection were used as controls. (A) Survival of IFNαR-/- mice post-challenge was monitored. (B) At 48 hours post-challenge, viruses in blood were quantified by qRT-PCR. The dashed line indicates the detection limit.

Because CHIKV 37997-mCherry is attenuated like the vaccine strain, we next tested if infection with CHIKV 37997-mCherry provides a protection similar to the vaccine strain (**Fig 5**). The IFNαR-/- mice that survived infection with 10 PFU to 10^3^ PFU of CHIKV 37997-mCherry or CHIKV 181/25 were challenged with 10 PFU of pathogenic CHIKV LR strain. All the mice pre-exposed to either CHIKV 37997-mCherry or CHIKV 181/25 survived (**Fig 5A**), although it was apparent sterilizing immunity was not induced in some animals. However, viral loads of challenge virus were limited compared to CHIKV LR replication in the control mice (**Fig 5B**), which demonstrated death in 100% of the mice within 4 days (**Fig 5A**).

**Fig 6:**
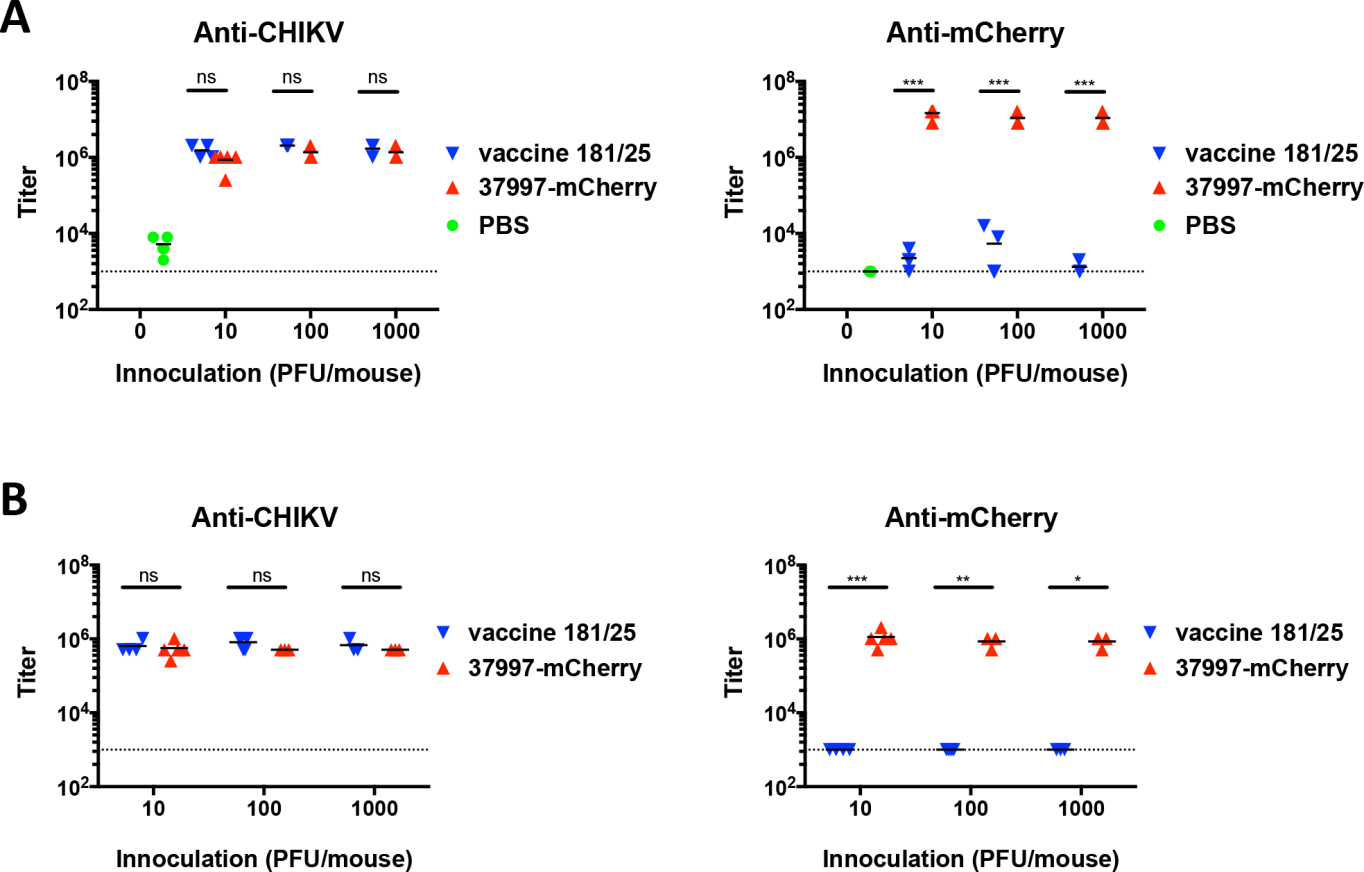
Antibody responses induced by vaccine strain 181/25 and 37997-mCherry. Anti-CHIKV (left) and anti-mCherry (right) total IgG antibody titers in surviving IFNαR-/- mice (A) at 3 weeks after CHIKV 181/25 and CHIKV 37997-mCherry infection and (B) at 11 days post-challenge with CHIKV LR were measured by end-point ELISA. The dashed lines indicate the detection limits. Statistical significance was determined by ANOVA with a Sidak’s multiple comparisons test (*P<0.0332; **P<0.0021; ***p<0.0002) (n = 3–5).

**Fig 7:**
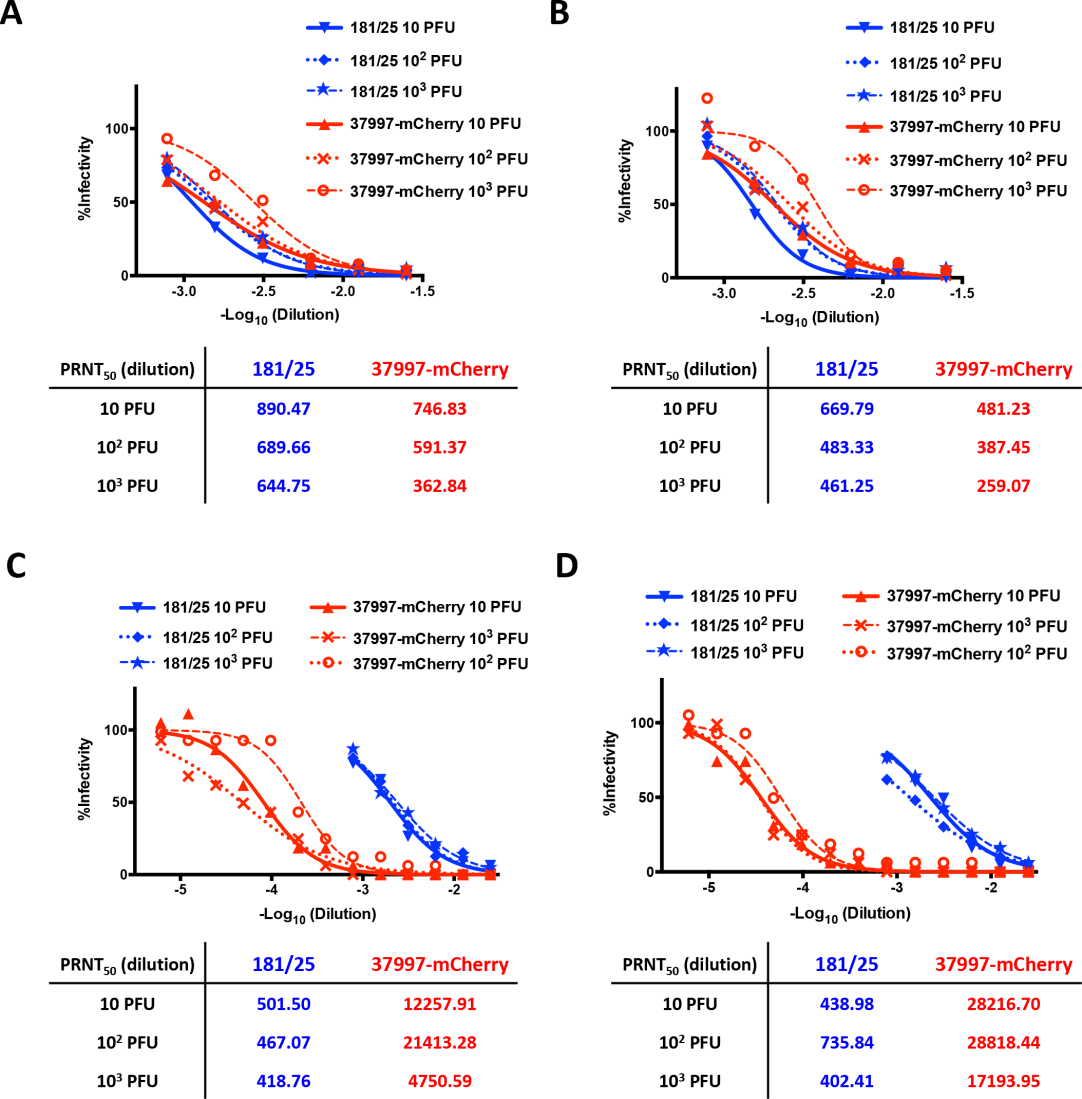
Neutralization of CHIKV by serum from infected IFNαR-/- mice. Infectivity of CHIKV vaccine strain 181/25 (A&B) or 37997-mCherry (C&D) was neutralized by serum from surviving IFNαR-/- mice **(A&C)** at 3 weeks after CHIKV 181/25 and CHIKV 37997-mCherry infection and **(B&D)** at 11 days post-challenge with CHIKV LR.

We next compared antibody responses in CHIKV 37997-mCherry *vs* CHIKV 181/25 infected IFNαR-/- mice before (**Fig 6A**) and after (**Fig 6B**) re-challenge with CHIKV LR. Infection with 10, 100 or 1,000 PFU of CHIKV 37997-mCherry and CHIKV 181/25 induced similar levels of antibodies against CHIKV in mice at 3 weeks post-infection and 11 days post-challenge with CHIKV LR. As expected, antibodies against mCherry were induced in mice infected with 37997-mCherry. Neutralizing activities of the antiserum in CHIKV 37997-mCherry and CHIKV 181/25 infected mice were next tested using a plaque reduction neutralization assay (**Fig 7**). Serum from CHIKV 37997-mCherry and CHIKV 181/25 vaccinated mice at both 3 weeks post-infection (**Fig 7A**) and 11 days post-re-challenge (**Fig 7B**) neutralized virus entry of CHIKV 181/25 at similar efficiency. Interestingly, serum from CHIKV 37997-mCherry vaccinated mice neutralized virus entry of CHIKV 37997-mCherry more efficiently than serum from CHIKV 181/25 vaccinated mice, with IC50s for the former ~30-fold higher than those for the latter **(Fig 7C and 7D)**. Serum from CHIKV 181/25 infected mice neutralized CHIKV 181/25 and CHIKV 37997-mCherry at similar efficiency (**Fig 7**), excluding the possibility that serum from CHIKV 181/25 infected mice only weakly neutralized CHIKV 37997-mCherry.

### CHIKV 37997-mCherry is attenuated in a chronic infection mouse model

**Fig 8:**
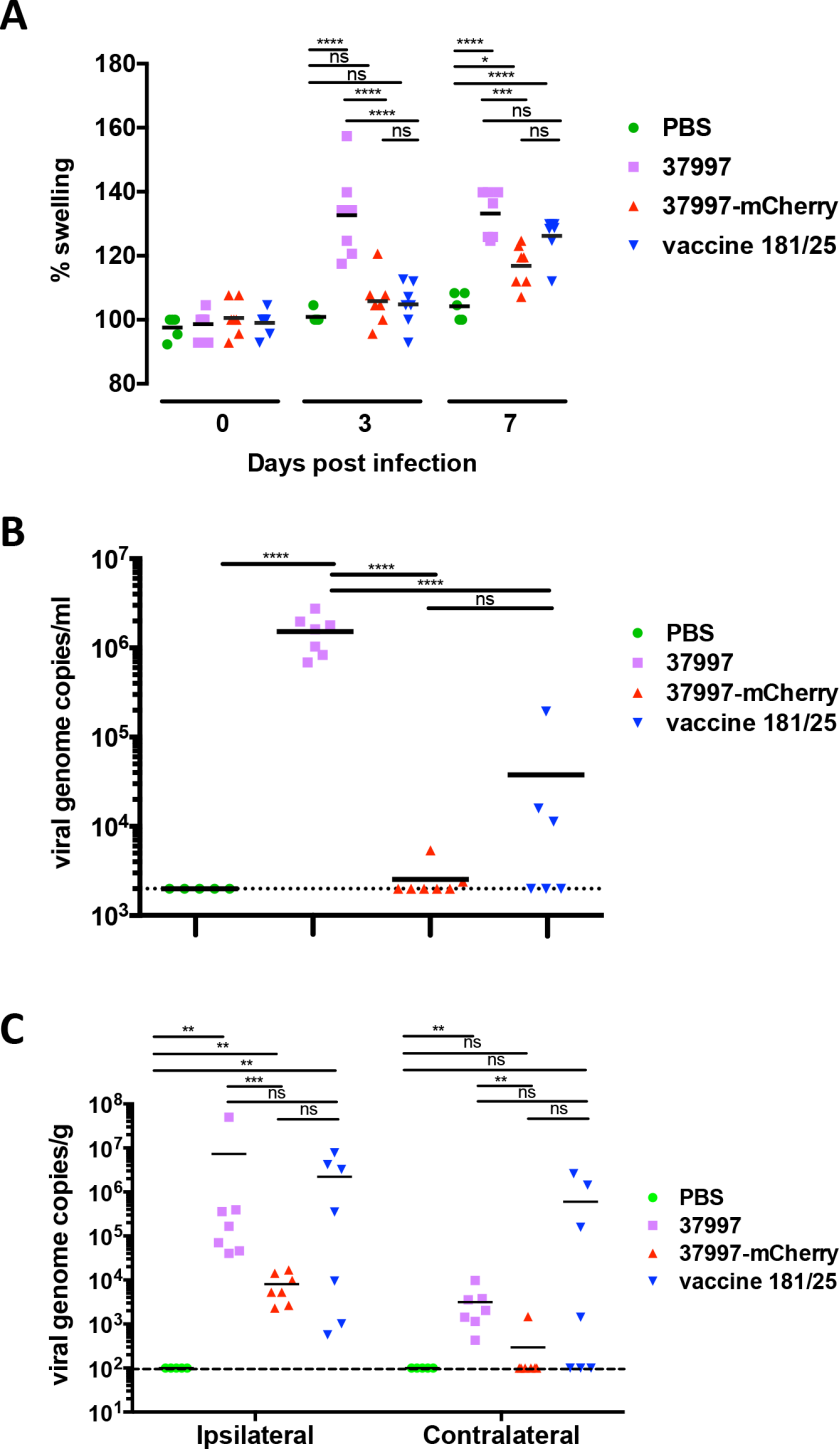
Attenuation of CHIKV 37997-mCherry in wild-type mice. Three weeks old C57BL/6 mice were injected with 10^3^ PFU of indicated viruses at left rear footpad. (**A**) Rear footpad sizes (width x height) were measured at indicated time points, (**B**) virus titers in blood were quantified by qRT-PCR at 3 days post-infection, and (**C**) virus titers in rear ankles were quantified by qRT-PCR at 28 days post-infection. The dashed lines indicate the detection limits. Statistical significance was determined by ANOVA with a Turkey’s multiple comparisons test (*P<0.0332; **P<0.0021; ***p<0.0002, ****p<0.0001) (n = 5–7).

After we found CHIKV 37997-mCherry was attenuated in the lethal mouse model of immunocompromised IFNαR-/- mice, we next compared its pathogenesis with the parental and vaccine strains in a chronic infection mouse model (**Fig 8**). Three weeks old wild-type mice were infected with 10^3^ PFU of CHIKV 37997, CHIKV 37997-mCherry or CHIKV 181/25 by injection in the rear footpad. As expected, CHIKV 37997 infection caused significant swelling of the footpads on the ipsilateral side at day 3 and day 7 post-infection (**Fig 8A**). In contrast, infection with CHIKV 37997-mCherry did not cause significant footpad swelling on day 3 and significantly less swelling than CHIKV 37997 on day 7 (**Fig 8A**). Tissue inflammation induced by infection of the vaccine strain CHIKV 181/25 in this chronic infection model was also attenuated to a similar level as CHIKV 37997-mCherry (**Fig 8A**). Consistently, plasma RNA viral loads at 3 days post-infection of CHIKV 37997-mCherry or CHIKV 181/25 were significantly lower than those in CHIKV 37997 infected mice (**Fig 8B**).

**Fig 9:**
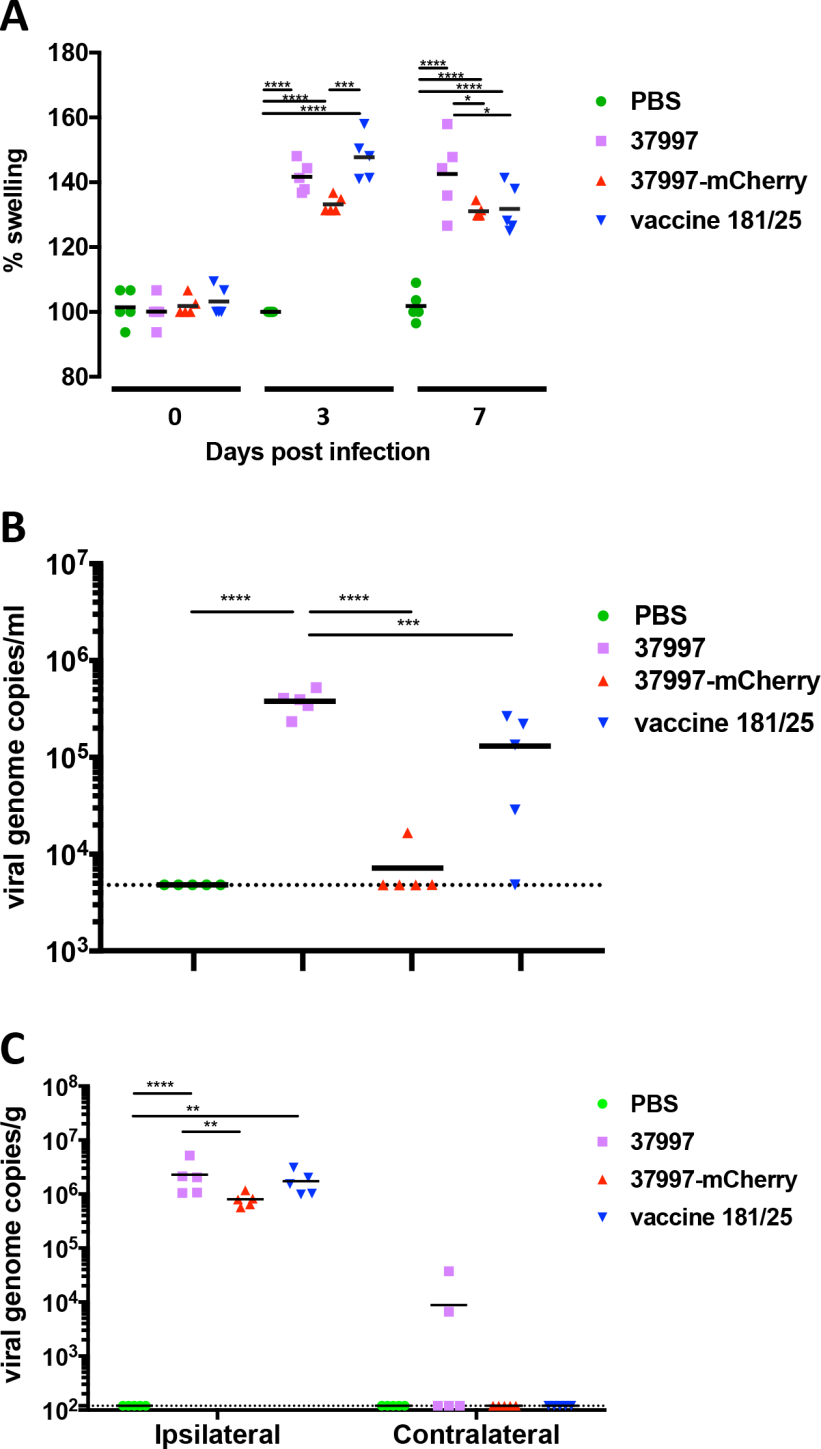
Attenuation of CHIKV 37997-mCherry at high dose in wild-type mice. Three week old C57BL/6 mice were injected with 10^6^ PFU of indicated viruses in the left rear footpad. (**A**) Rear footpad sizes (width x height) were measured at indicated time points, (**B**) virus titers in blood were quantified by qRT-PCR at 3 days post-infection, and (**C**) virus titers in rear ankles were quantified by qRT-PCR at 28 days post-infection. The dashed lines indicate the detection limits. Statistical significance was determined by ANOVA with a Turkey’s multiple comparisons test (*P<0.0332; **P<0.0021; ***p<0.0002, ****p<0.0001) (n = 5).

At 28 days post-infection, in the chronic phase of CHIKV infection, persistent viral RNA in ankle tissues was compared (**Fig 8C**). As we, and other researchers, have reported before [16, 20], CHIKV persists in both the ipsilateral and contralateral ankles after CHIKV 37997 infection. In contrast, viral RNA in the ankles on the contralateral side of CHIKV 37997-mCherry infected mice were not significantly higher than the assay limit of detection, and viral loads in ankles on the ipsilateral side were significantly lower than those in parental CHIKV 37997 infected mice (**Fig 8C**). In vaccine strain CHIKV 181/25 infected mice viral loads varied extensively between individual mice in ankles of both sides (**Fig 8C**), consistent with the large variation of plasma viral load in CHIKV 181/25 infected mice (**Fig 8B**). In some CHIKV 181/25 infected mice, virus replicated and persisted at high loads relative to those in CHIKV 37997-mCherry infected mice, although no statistical significant difference could be demonstrated between the two groups. Similar attenuation of CHIKV 37997-mCherry at a dose as high as 10^6^ PFU was observed in 3-weeks old mice (**Fig 9**).

### Infection with CHIKV 37997-mCherry induced a strong antibody response against mCherry

**Fig 10:**
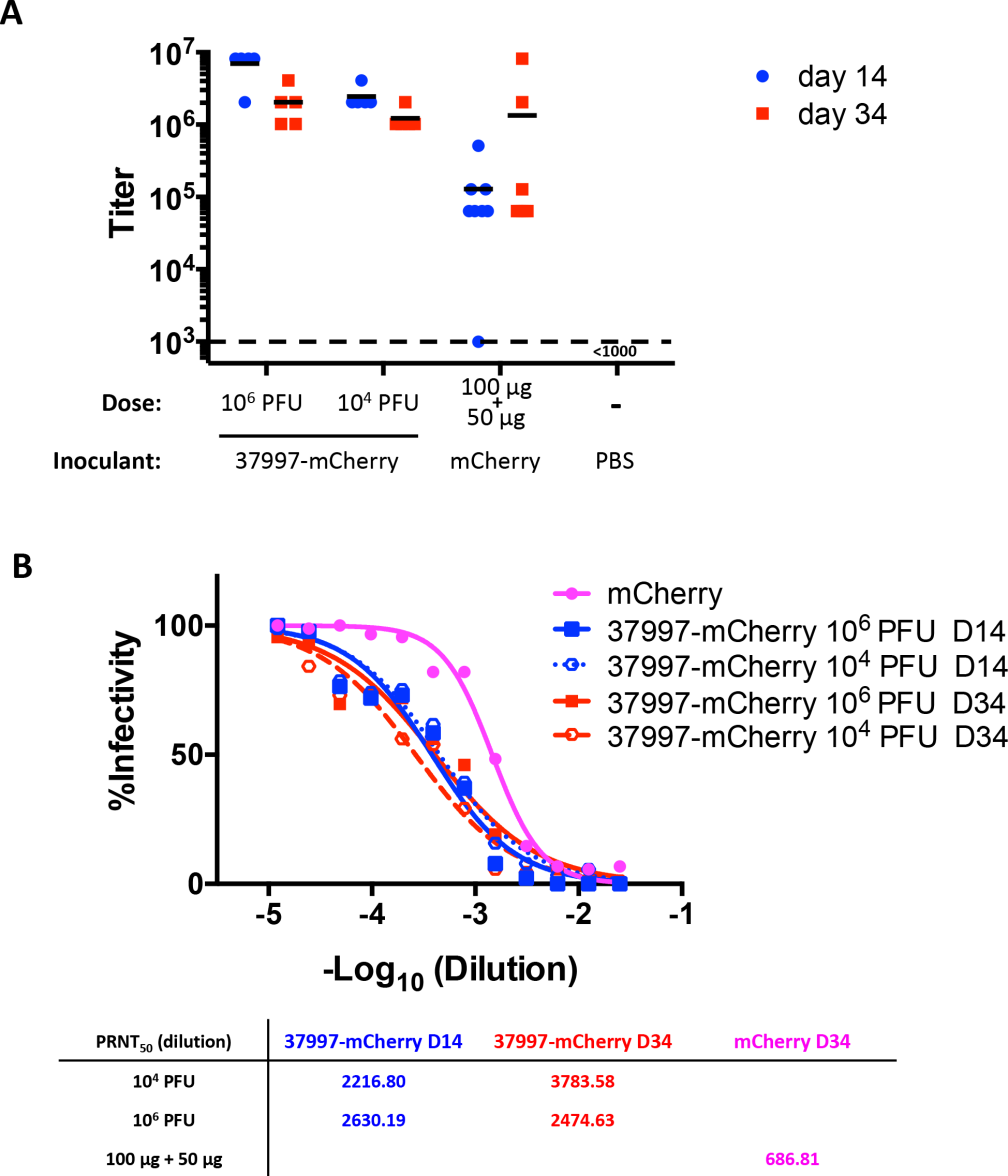
CHIKV 37997-mCherry infection induces antibody responses against mCherry. Eight weeks old C57BL/6J mice were infected with indicated amount of CHIKV 37997-mCherry (5 mice per group) or prime immunized with 100 μg of mCherry protein followed by boosting with 50 μg of mCherry protein 2 weeks later (8 mice). (A) Titers of anti-mCherry antibodies induced in mice at 14 and 34 days post-infection were measured by end-point ELISA. The dashed line indicates the detection limit. (B) Neutralization of CHIKV 37997-mCherry by serum from immunized mice.

We observed presentation of mCherry on the surface of chimeric CHIKV 37997-mCherry in a repeating pattern with 240 copies of mCherry on each virion. Strong anti-CHIKV responses were induced by infection of CHIKV 37997-mCherry, so we next tested if strong antibody responses against mCherry could be induced by CHIKV 37997-mCherry. In adult mice, infection with 10^4^ or 10^6^ PFU of CHIKV 37997-mCherry induced high antibody responses against mCherry when assessed at day 14 post-infection and the high levels of antibody lasted for at least 34 days (**Fig 10A**). Immunization with 100 μg of purified mCherry protein in complete Freud’s adjuvant induced antibodies against mCherry at more than 20 fold lower level than induced by CHIKV 37997-mCherry infection at day 14 (**Fig 10A**). Boosting with 50 μg of mCherry enhanced antibody responses to the level induced by CHIKV 37997-mCherry infection in only 2 of 7 mice (**Fig 10A**). Interestingly, serum from mCherry immunized mice could neutralize CHIKV 37997-mCherry virus entry, although at a lower efficiency than the serum from CHIKV 37997-mCherry infected mice (**Fig 10B**). This is consistent with the results that the serum from CHIKV 37997-mCherry infected IFNαR-/- mice neutralized CHIKV 37997-mCherry at about a ~30-fold higher efficiency than the serum from CHIKV 181/25 infected IFNαR-/- mice (**Fig 7C & 7D**).

## Discussion

CHIKV is the most common alphavirus infecting humans–with millions of individuals infected during the 2000s, resulting in thousands of deaths [2]. Currently there are no licensed vaccines or treatments for CHIKV infection. An attenuated live vaccine strain (CHIKV 181/25) was developed and reported to be safe, highly immunogenic and produced well-tolerated side effects in a phase II clinical trial [21–23]. Two point mutations at I12 and R82 in the E2 glycoprotein of CHIKV 181/25 are responsible for its attenuation of acute disease in mice [24, 25]. Acquisition of R82 in E2 enhances the affinity of CHIKV 181/25 particles for glycosaminoglycans (GAGs) *in vitro* [26], which may limit the capacity of CHIKV to disseminate from early sites of primary replication *in vivo* [24, 27]. A recent study reported that R82 in E2 also renders CHIKV 181/25 vulnerable to neutralization by antibodies targeting E2 domain B and therefore more likely to be cleared [28]. In our study, CHIKV 37997-mCherry was more efficiently neutralized by serum from CHIKV 37997-mCherry infected mice than from CHIKV 181/25 infected mice **(Fig 7)**. This may explain the quick clearance of CHIKV 37997-mCherry in blood and ankle tissues **(Figs 8 & 9)** and therefore the attenuation of CHIKV 37997-mCherry *in vivo*. Interestingly, we found antiserum against mCherry neutralized virus entry of CHIKV 37997-mCherry although at a lower efficiency than the antiserum raised in CHIKV 37997-mCherry infected mice **(Fig 10)**. mCherry proteins displayed on the surface of CHIKV 37997-mCherry particle was not expected to mediate either the virus-receptor interaction or membrane fusion during virus entry. How anti-mCherry antibodies neutralize CHIKV 37997-mCherry warrants further studies. We can speculate that binding of antibodies to mCherry may limit the flexibility of E2 domain B and therefore prevent the activation of the fusion loop for virus entry. This additional neutralizing epitope and the presence of anti-mCherry antibodies provides the likely explanation for the ~30-fold more efficient neutralization of CHIKV 37997-mCherry by the antiserum raised in CHIKV 37997-mCherry infected mice compared to antiserum raised in CHIKV 181/25 infected mice, and therefore the attenuation of CHIKV 37997-mCherry **(Fig 7)**. Whether or not chimeric CHIKV virus carrying other foreign antigens is attenuated depends on whether the foreign antigens are presented as neutralizing epitopes.

Reversion of attenuating mutations was reported in CHIKV 181/25 infected wild-type mice [24, 25]. The insertion of mCherry between E3 and E2 in CHIKV 37997 genome may not be so easy to revert compared to reversion of single amino acid mutation. This could explain the large variation of virus titers in blood and ankle tissues in mice infected with CHIKV 181/25 compared to consistent low virus titers in mice infected with CHIKV 37997-mCherry **(Figs 8 & 9)**. Future studies on virus stability *in vitro* and *in vivo* as well as *in vivo* tropism of CHIKV 37997-mCherry are needed to understand the dynamics of the virus populations.

On each chimeric CHIKV 37997-mCherry virion, 240 copies of mCherry are presented in a repetitive pattern between viral spikes **(Fig 2)**. The high density of mCherry can be easily recognized by B cells and, therefore, activate B cells efficiently. Consistently, a single infection of CHIKV 37997-mCherry induced long-lasting and high titer antibody responses against mCherry that can only be reached after boosting in mice conventionally immunized with mCherry proteins **(Fig 10)**. Our results suggest that fusions to E2 may be a promising vaccine platform. Fusion of foreign antigens to E2 generates replication-competent chimeric virus able to present these antigens in a repetitive array on virion surface [9, 10]. Live attenuated chimeric aphaviruses are able to induce strong antibody responses against the foreign antigen as well as anti-alphavirus response. Recently, a study was reported using CHIKV virus-like particles as a vaccine platform to present NANP repeats from the circumsporozoite protein (CSP) of the *Plasmodium falciparum* malaria parasite [11]. Similarly the dense array of NANP repeats on VLPs induced strong antibody responses that protected mice from malaria infection. In that study NANP repeats of 58 amino acid were inserted on E3 and E2 separately. In our study, a much larger antigen, mCherry (236 amino acid) was fused to E2 with little effect on E3 cleavage efficiency, which permits infectivity of the chimeric virus. Virus replication generates viral RNA replication intermediates and viral RNA that strongly activate host innate immune responses, which may explain the strong immunogenicity of our replication-competent chimeric virus[29].

In summary, we report an attenuated mCherry tagged replication-competent CHIKV that may suggest an additional risk mitigation should an individual be accidentally infected. In addition our work suggests similar chimeric replication-competent alphaviruses can serve as vaccines against other pathogens and diseases, in addition to the chimeric VLP-based vaccines.

## Supporting information

S1 Fig Intact insertion of mCherry in gradient-purified CHIKV 37997-mCherry virus. Intact insertion of mCherry in CHIKV 37997-mCherry virus in the supernatant of cells electroporated with *in vitro* transcribed viral genomic RNA (passage 0) and in the gradient-purified CHIKV 37997-mCherry expanded *in vitro* was confirmed by RT-PCR. Viral RNA was extracted from the virus followed by RT-PCR with the primers flanking the E3-mCherry-E2 coding region. The plasmid that carries the whole genome of CHIKV 37997-mCherry was used as the control for PCR.
(TIF)
