# Supplementary figures and images for: An attenuated replication-competent chikungunya virus with a fluorescently tagged envelope
Source: PLoS Negl Trop Dis. 2018 Jul 31;12(7):e0006693. doi: 10.1371/journal.pntd.0006693 (PMC6086482; doi:10.1371/journal.pntd.0006693)

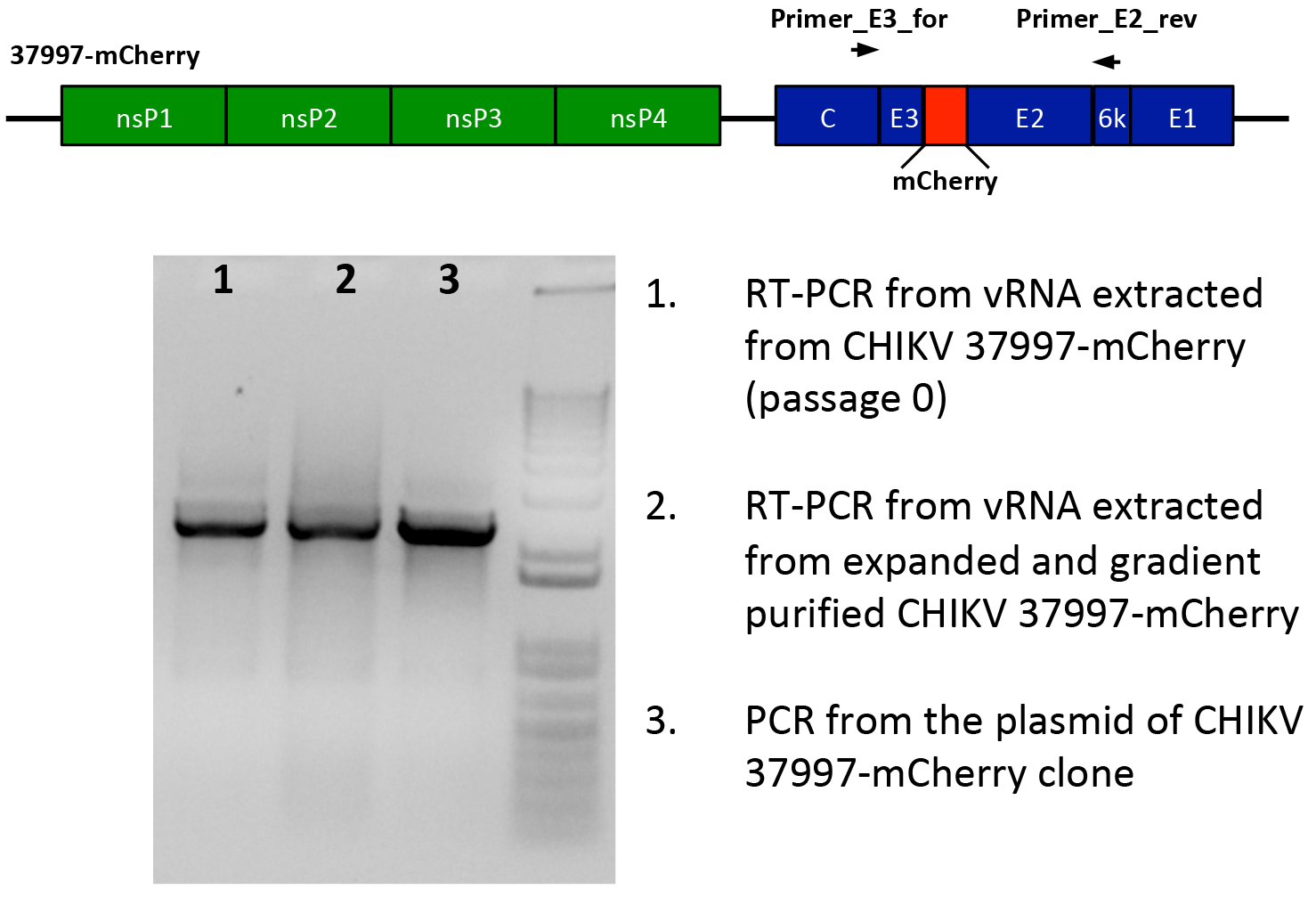

Supplement: S1 Fig — Intact insertion of mCherry in CHIKV 37997-mCherry virus in the supernatant of cells electroporated with in vitro transcribed viral genomic RNA (passage 0) and in the gradient-purified CHIKV 37997-mCherry expanded in vitro was confirmed by RT-PCR. Viral RNA was extracted from the virus followed by RT-PCR with the primers flanking the E3-mCherry-E2 coding region. The plasmid that carries the whole genome of CHIKV 37997-mCherry was used as the control for PCR. (TIF) [file pntd.0006693.s001.tif]
